# The Coexistence Relationship Between Plants and Soil Bacteria Based on Interdomain Ecological Network Analysis

**DOI:** 10.3389/fmicb.2021.745582

**Published:** 2021-12-07

**Authors:** Wei Cong, Jingjing Yu, Kai Feng, Ye Deng, Yuguang Zhang

**Affiliations:** ^1^Key Laboratory of Biodiversity Conservation of National Forestry and Grassland Administration, Research Institute of Forest Ecology, Environment and Protection, Chinese Academy of Forestry, Beijing, China; ^2^CAS Key Laboratory for Environmental Biotechnology, Research Center for Eco-Environmental Sciences, Chinese Academy of Sciences (CAS), Beijing, China

**Keywords:** interdomain ecological networks, plant-bacteria coexistence, topological structure, SparCC method, 16S rRNA high-throughput sequencing

## Abstract

The relationship between plants and their associated soil microbial communities plays a crucial role in maintaining ecosystem processes and function. However, identifying these complex relationships is challenging. In this study, we constructed an interdomain ecology network (IDEN) of plant–bacteria based on SparCC pairwise associations using synchronous aboveground plant surveys and belowground microbial 16S rRNA sequencing among four different natural forest types along the climate zones in China. The results found that a total of 48 plants were associated with soil bacteria among these four sites, and soil microbial group associations with specific plant species existed within the observed plant–bacteria coexistence network. Only 0.54% of operational taxonomy units (OTUs) was shared by the four sites, and the proportion of unique OTUs for each site ranged from 43.08 to 76.28%, which occupied a large proportion of soil bacterial community composition. The plant–bacteria network had a distinct modular structure (*p* < 0.001). The tree *Acer tetramerum* was identified as the network hubs in the warm temperate coniferous and broad-leaved mixed forests coexistence network and indicates that it may play a key role in stabilizing of the community structure of these forest ecosystems. Therefore, IDEN of plant–bacteria provides a novel perspective for exploring the relationships of interdomain species, and this study provides valuable insights into understanding coexistence between above-ground plants and below-ground microorganisms.

## Introduction

Understanding the associations between plants and soil microbes has been a key issue in ecology. Complex interactions between plants and soil microbes can influence their community structure, plant diversity, and ecosystem function (Rudgers et al., 2020; Yang et al., 2021). For example, plant roots impact the composition of rhizosphere microbial communities by secreting organic carbon and antibacterial substances (Sasse et al., 2017). Microorganisms can transform soil nutrients to facilitate plant absorption (Averill et al., 2019; Hestrin et al., 2019), improve plant tolerance to environmental stress (Pieterse et al., 2014; Trivedi et al., 2020), and booster plant disease resistance (Fitzpatrick et al., 2018; Giauque et al., 2018). In addition, soil microbial diversity is important to maintaining plant productivity to stabilize ecosystem functions (Chen et al., 2020). Nevertheless, exploring the intricate relationships of aboveground–belowground ecosystems remains a challenge.

The network analysis is one of the important ways to help explain the relationship of complex systems. Ecological network analysis can elucidate community assembly, predict stability of the community structure and reveal ecological processes, and provide insight into detecting keystone species, as well as the complex interactions of species (Layeghifard et al., 2016). Interactive relationships have been intensively observed among different organisms by using network analysis, including host–parasite networks (Fortuna et al., 2010), plant–pollinator networks (Bascompte et al., 2003; Dormann et al., 2009), and plant–frugivore networks (Bascompte et al., 2003). However, most studies on plant–microbe networks have focused on the relationship between plants and fungi. For example, Toju et al. (2015) observed antinestedness in plant–fungus networks, which was considered to promote species coexistence in plant partner networks and was more complex variable than plant–pollinator and plant–seed networks. Literatures on the study of the coexistence of aboveground plants and belowground soil bacteria are still few.

To explore the relationships between plants, bacteria, and archaea, Feng et al. (2019) constructed interdomain ecological networks (IDENs) based on abundance datasets and identified particular topological features of the regional IDEN and endemicity of geographical distribution between plants and microorganisms. The IDEN is constructed by pairwise association calculations (i.e., SparCC and SPIEC-EASI) *via* microbial high-throughput sequencing data and plant distribution survey data, and it deduces associations between plants and microbes by utilizing correlation-based approaches. The IDEN workflow provides a novel perspective to understand different domain species associations. Feng et al. (2019) constructed the IDEN analysis pipeline and is a useful workflow to explore relationships between species in natural ecosystems.

Forest ecosystems have abundant biodiversity and complex ecological networks, which dominate the carbon cycle process of terrestrial ecosystems, maintain the structural and functional stability of the ecosystem, and play an irreplaceable role in improving the ecological environment. To understand the coexistence of plants and soil bacteria, we selected typical natural forest from different climatic zones, with different forest types and minimal human disturbance, and constructed IDEN based on SparCC pairwise association calculations by using plant distribution survey data and 16S rRNA sequencing data (Feng et al., 2019). Our findings showed that asymmetric specialization, modularity, and non-nestedness were obvious topological structural characteristics for the plant–bacteria network, and soil bacteria had distinct geographic distributions and showed preferences for particular plant species. The tree *Acer tetramerum* is the network hub in the warm temperate coniferous and broad-leaved mixed forests coexistence network, and it may play a key role in maintaining the stability of the community structure in these forest ecosystems.

## Materials and Methods

### Site Description and Soil Sampling

In this study, we selected four typical natural forest types along climatic zones in China, including temperate coniferous forest, warm temperate coniferous and broad-leaved mixed forest, subtropical broad-leaved forest, and tropical rain forest. The four forest types were located in the Kanas national nature reserve, Xinjiang Uygur Autonomous Region (KNS; E86.97, N48.89), Xiaolong Mountain national nature reserve, Gansu Province (GS; E106.27, N34.24), Mulinzi national nature reserve, Hubei Province (MLZ; E109.64, N29.65), and Bawangling national nature reserve, Hainan Province (HN; E109.66, N19.06). At each site, 17 representative plots of 20 m × 20 m were selected. Soil samples were collected from 16 surface soil cores (0–10 cm) with more than 1-m distance at each plot. All soil samples in the same plot were then mixed as one sample. A total of 68 plots were sampled in this study. Plant communities were surveyed by recording plant species, height, and counts of all woody plant exceeding 1.0 cm in diameter at breast height for each plot.

### Soil Microbial DNA Extraction, Illumina Sequencing, and Data Analyses

Soil microbial DNA was extracted using the PowerSoil kit (Qiagen, Germany) following protocol instructions. Crude DNA was purified by gel electrophoresis and measured by using an ND-1000 spectrophotometer (Nanodrop Inc.). DNA concentration was quantified by using FLUOstar Optima (BMG Labtech, Jena, Germany) (Ahn et al., 1996). Purified DNA was amplified at the V3–V4 region of the 16S rRNA gene with the primer pair 338F (5′-ACTCCTACGGGAGGCAGCAG-3′) and 806R (5′-GGACTACHVGG GTWTCTAAT-3′) (Mori et al., 2014), and sequenced on an Illumina MiSeq sequencing platform (Majorbio, China).

Raw sequencing data were separated according to corresponding barcodes. Low-quality and ambiguous sequences were discarded. FLASH was used to integrate reads into a whole sequence (Tanja and Salzberg, 2011). The operational taxonomy units (OTUs) were formed at 97% similarity level by using Usearch (Edgar, 2013), and the taxonomy for each OTU was determined using RDP classifier (Wang et al., 2007). A total of 20,000 sequences for each sample remained for further analysis. The raw data were processed using a Galaxy pipeline^1^.

### Interdomain Ecological Network Construction

Interdomain ecology network data analysis was completed on the Galaxy-IDENAP platform^2^ (Feng et al., 2019). SparCC, a correlation-based approach, was used for infer potential association of plants and microorganisms (Friedman and Alm, 2012). We removed data with absolute value of correlation coefficient smaller than 0.3. SparCC results were also filtered, and the data were kept according to *p* <0.05 value. Network topological structures, module detection, and random network were analyzed based on the observed IDEN. We used simulated annealing to determine the compartmentalization of the observed IDEN (Guimera and Amaral, 2005). Random networks were created by a null model, which generated 100 rewired networks and compared the significance of topological features for the observed IDEN and random networks (Deng et al., 2012). The observed network was visualized using the Gephi (0.9.2) (Bastian et al., 2009). Venn diagram was analyzed based on Venny’s online (Oliveros et al., 2007–2015). A one-sample *t* test was used to measure the significance between observed and random networks. All the analyses were conducted using “bipartite” and “ggplot2” packages in R Studio (v.3.4.3).

## Results

### Interdomain Ecology Network Topological Characterization of Plant–Bacteria Associations

We constructed four IDENs using high-throughput sequencing and plant richness datasets (Table 1). Results exhibited that plant–bacteria associations were different among the four forest types. The plant–bacteria associations consisted of 4 plants and 313 bacterial OTUs with 342 links in KNS, 16 plants and 915 bacterial OTUs with 2,353 links in GS, 9 plants and 571 bacterial OTUs with 1,144 links in MLZ, and 20 plants and 647 bacterial OTUs with 1,645 links in HN. Although plant–bacteria associations were few in KNS, the connectance (0.273), web asymmetry (−0.975), and cluster coefficient (0.297) were the highest compared to the other three forest types. The observed plant–bacteria associations had 27.3% possible links. We observed non-nested structure in the four plant–bacteria networks. In addition, modularity based on simulate annealing was the highest in KNS (0.570). KNS, MLZ, and HN had four modules, whereas GS had five modules.

**TABLE 1:** Network topological structural properties of the plant–bacteria network.

	KNS	GS	MLZ	HN
No. plants	4	16	9	20
No. microbes	313	915	571	647
Total links	342	2353	1144	1645
Connectance	0.273	0.161	0.223	0.127
Web asymmetry	−0.975	−0.966	−0.969	−0.940
Links per species	1.079	2.527	1.972	2.466
Cluster coefficient	0.297	0.122	0.235	0.107
Nestedness	41.853	28.360	32.762	32.771
Weighted nestedness	0.481	0.380	0.381	0.190
Specialization asymmetry	0.756	0.488	0.654	0.552
Modularity (simulated annealing)	0.570	0.353	0.382	0.413
No. modules	4	5	4	4

*KNS, Kanas; GS, Gansu; MLZ, Mulinzi; HN, Hainan.*

Observed and random networks were compared by rewiring 100 networks (Supplementary Table 1). The one-sample *t* test showed significant differences (*p* <0.05) in topological structures of the four plant–bacteria networks, such as asymmetric specialization and modularity (*p* <0.001). The checkerboard scores for plants showed no significant difference existed between the observed network and random network in KNS (*t* = 1.787, *p* = 0.077). Therefore, the plant–bacteria network had a distinct modular structure and non-randomness.

### Plant–Bacteria Association Geographical Distribution Characteristics

**FIGURE 1:**
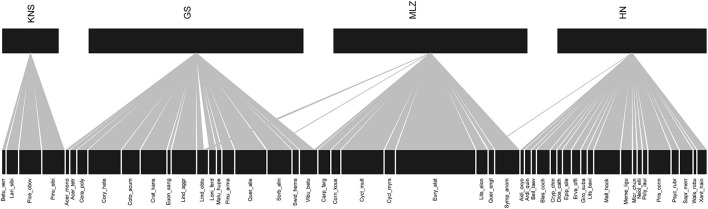
Geographical distribution of the plants associated with bacteria. The widths of rectangles were the abundance of plant species. KNS, Kanas; GS, Gansu; MLZ, Mulinzi; HN, Hainan.

A total of 48 plants were associated with soil bacteria at the four sites (Figure 1). *Lindera obtusiloba*, *Quercus aliena*, and *Viburnum betulifolium* were identified in GS and MLZ, but *Q. aliena* and *V. betulifolium* were only involved in the plant–bacteria association network for GS. Interestingly, *Litsea elongata* was associated with the MLZ plant–bacteria network despite it also being found in MLZ and HN. Therefore, our results indicated that plant species have a strong regional distribution pattern among four different forest types.

**FIGURE 2:**
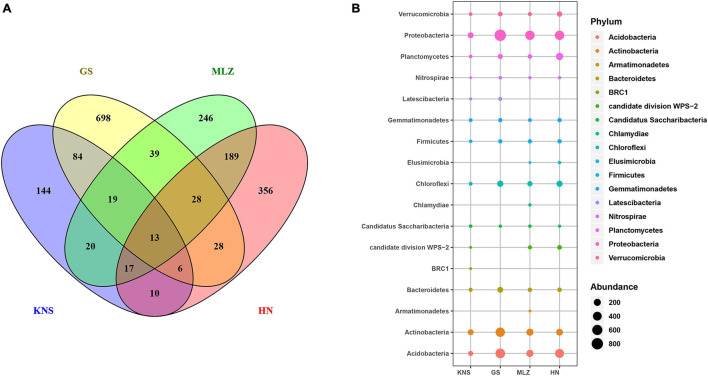
Geographical distribution of soil bacteria in four forest types (KNS = Kanas, GS = Gansu, MLZ = Mulinzi, HN = Hainan). **(A)** A Venn diagram of soil bacteria in four forest types, illustrating the shared and exclusive number of OTUs. **(B)** The composition of soil bacterial communities in four forest types at the phylum level.

In order to identify whether soil bacteria have the same geographic distribution pattern as plants, a Venn diagram was used to illustrate the shared and exclusive OTUs at the four sites (Figure 2A). The unique bacterial OTUs accounted for 46.01, 76.28, 43.08, and 55.02% of the KNS, GS, MLZ, and HN, respectively. The percentage of OTUs shared by the two sites ranged from 1.05 to 18.37%. Note that shared OTUs for four sites only amounted to 13 and accounted for only 0.54%. Among the relatively close geographic distances sites had relatively high shared OTUs; for example, MLZ and HN shared 189 OTUs and accounted for 18.37%. Among the relative far geographic distance sites had a low shared OTUs, for example, KNS and HN shared only 10 OTUs and accounted for 1.05%. We also investigated soil bacterial group associations with specific plant species at the taxonomic level within the observed plant–bacteria coexistence network (Figure 2B and Supplementary Figure 1). For example, BRC1 was found only in KNS and was in connection with *Betula pendula*; Armatimonadetes and Chlamydiae, found only in MLZ, were in connection with *Cyclobalanopsis multinervis*. Therefore, soil bacteria showed geographical distribution characteristics and showed preference for specific plant species among these natural forest types.

### Plant–Bacteria Association Differences in Interdomain Ecology Network Modules

**FIGURE 3:**
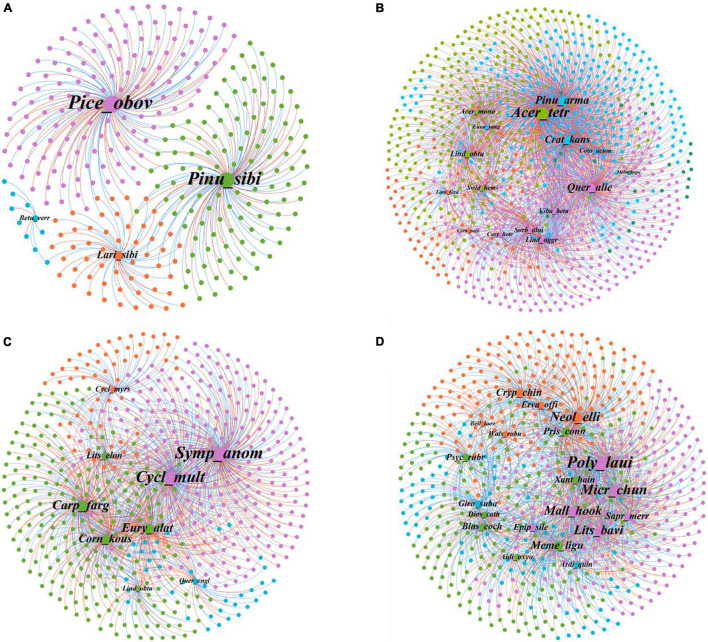
The plant–bacteria network architecture. **(A)** Kanas (KNS); **(B)** Gansu (GS); **(C)** Mulinzi (MLZ); **(D)** Hainan (HN). For each network, plants and microorganisms within the same module are indicated in the same color while different colors represent different modules. Node size are associated with node degree.

The connection of plant–bacteria nodes and potential ecological connections was explored using module separation analysis (Figure 3 and Supplementary Table 2). There were four modules in KNS, and each module contains only one plant. Module 4 with *Picea obovata* had 133 OTUs, which is the largest number of nodes observed (147 nodes). There were five modules in GS, and module 1 had the greatest number of plants and OTUs (5 plants and 303 OTUs), whereas module 5 had the least number of plants and OTUs (1 plant and 24 OTUs). Notably, *A. tetramerum* in module 3 had the most nodes across GS, were classified as network hubs (*Z*_*i*_ = 12.89, *p*_*i*_ = 0.67), which play a crucial role in GS networks. There were four modules in MLZ. Module 1 had the most nodes (240 nodes), whereas module 3 has the largest number of plants and OTUs (5 plants and 222 OTUs). There were 20 unique plant species among the four modules in HN. Module 4 contained the greatest diversity of plants, including *Aidia oxyodonta*, *Blastus cochinchinensis*, *Diospyros cathayensis*, *Memecylon ligustrifolium*, *Prismatomeris connata*, *Psychotria rubra*, and *Xanthophyllum hainanense*. Module 3 contained 226 OTUs and *Polyalthia lauii* had the largest number of nodes (178 nodes). In addition, *Beilschmiedia laevis* in module 1 was classified as a peripheral species (*Z*_*i*_ = 1.63, *p*_*i*_ = 0.42), meaning it is less important in HN networks.

Further analysis of the related soil bacterial groups among modules at each site showed that there are differences in bacterial abundance between different modules (Supplementary Figure 2). For example, among the four modules of KNS, BRC1 existed only in module 1, and Nitrospirae existed only in module 4. Candidatus Saccharibacteria existed only in module 2 of the GS site, Armatimonadetes existed only in module 1 of the MLZ site, and Candidatus Saccharibacteria and Nitrospirae existed only in module 1 of the HN site. Therefore, species within the same module were found to be more closely related than species within different modules.

## Discussion

We used the IDEN analysis to explore the complex interrelationships between underground soil bacteria and aboveground plants and calculated a set of measures to describe common topological properties (such as connections, specialization asymmetry, cluster coefficient, nestedness, and modularity) in network analysis. The characteristics of the network structure facilitated the comparison of complex data from different ecosystems (Barberán et al., 2014). For example, specialization asymmetry may imply that plants are connected with microbial specialists. Our results showed that Armatimonadetes was connected only with *C. multinervis* in GS, and BRC1 was connected only with *B. pendula* in KNS. Moreover, the higher value of checkerboard scores for the plant–bacteria network in KNS indicated that plants are highly specific to microorganisms, presumably due to low richness of plants observed at the KNS plot. Bascompte et al. (2003) identified that asymmetrical and non-random patterns provide pathways that facilitate the presence of rare species and biodiversity. Nestedness is a pattern that specialists interact with a subset of the generalists in species interaction networks (Staniczenko et al., 2013). The present study showed that nestedness could reduce competition, increase biodiversity, and determine structural stability (Bastolla et al., 2009; Bruno et al., 2020; Santos and Sampaio, 2021). In plant–pollinator networks, a highly connected and nested architecture increases the persistence of pollinators after disturbance (Rohr et al., 2014; Geslin et al., 2017). Moreover, the nestedness was supported in the plant–fungus network and regional IDEN of plant–bacteria (Toju et al., 2014; Feng et al., 2019). It is worth noting that a non-nested structure was observed in each IDEN in our study. Similar observations by Feng et al. (2019), this may be a unique feature of local IDEN. Bastolla et al. (2009) showed nestedness is probably related to other properties of the network structure. Moreover, nestedness could be affected by climate change such as mean annual precipitation and temperature (Dalsgaard et al., 2013; Trøjelsgaard and Olesen, 2013). The variation of nestedness was better explained by the extent of host–plant overlap among partner species in plant–fungus networks (Toju et al., 2014). Staniczenko et al. (2013) found that non-nested structures indicate that species preferences are partitioned to reduce competitive opportunities. Further research is needed for understanding variation in nested structures among regional networks or local networks and pairwise comparisons (e.g., macroorganism–macroorganism and macroorganism–microorganism).

Some evidences showed that distribution patterns of plants are related to abiotic and biotic factors. Huang et al. (2021) showed that the geographical distribution range and latitude distribution range of plants are influenced by climate variability and extremes. Furthermore, plant range expansion was also found to be determined by belowground biota (Wisz et al., 2013). In this study, a total of 48 plants associated with microorganisms were observed. Plants and microorganisms showed significant differences in their geographical distribution patterns. For example, Armatimonadetes and Chlamydiae were connected with *C. multinervis*, which was found only in MLZ. This was seen in the interactions of plants with their associated antagonistic biotic (Engelkes et al., 2008). In addition, plant competition and community dynamics can be influenced by interactions between plants and soil microbes (Bever et al., 2012; Kandlikar et al., 2019). Likewise, there are a number of ways in which soil microbial community composition can be driven by plants. For instance, tree species identity can direct effects on bacterial community composition (Prada-Salcedo et al., 2020). Some soil microbes can form close relationships with particular plant species (Leff et al., 2018). This may be partly responsible for the different microenvironments formed by various vegetation types to adapt to the growth of different microbial groups (Faoro et al., 2010). On the other hand, the release of root exudates can also indirectly affect specific microbes and plant species associations (Sasse et al., 2017). In addition, geographical isolation and dispersal limitation may influence the distribution of microbial communities (Whitaker et al., 2003; Zhou and Ning, 2017).

The network analysis identified key species that may play an important role in maintaining community stability. Previous studies found that the composition and function of communities changed when keystone taxa were removed (Banerjee et al., 2018). Here, we found many plants belonging to module hubs. Particularly, *A. tetramerum* is a network hub in GS, indicating hubs species should preferentially get high conservation (Olesen et al., 2007). Modularity could reflect phylogenetic clustering of closely related species and niche overlap; higher modularity means more specialization (Olesen et al., 2007). Species within the same module interact with each other more frequently than between modules (Guimerà and Nunes Amaral, 2005). Fluctuation of taxa within a module is unlikely to spread to taxa in other modules when disturbance occurs (Hernandez et al., 2021). We observed an uneven distribution of nodes in each module. To be specific, *P. obovata* (temperate coniferous forests), *A. tetramerum* (warm temperate coniferous and broad-leaved mixed forests), *Symplocos anomala* (subtropical broad-leaved forests), *P. lauii* (tropical rain forests) have more nodes or are highly associated with microorganisms, showing that these may be associated with specific ecological functions and have similar responses to environmental perturbation. Studies have shown that the relative contribution of modularity differs in the type of interaction; for instance, modularity is usually higher in antagonistic interactions than observed in mutualism network (Thébault and Fontaine, 2010). Furthermore, several studies have reported the importance of climatic factors to modularity; for instance, modularity decreased with climate warming in seed-dispersal networks (Takemoto and Kajihara, 2016) and increased with rainfall seasonality in food web (Takemoto et al., 2014). Takemoto and Kajihara (2016) found that a decrease in modularity due to human activities was observed in food webs.

In this study, the relationships between plants and microorganisms were explored using the SparCC correlation analysis method based on IDENP (interdomain ecological network analysis pipeline). By comparing the similarity and differences of IDEN among different forest types, we found that the local IDEN showed particular structural properties and non-randomness, including asymmetric, specialization, non-nestedness and modularity, whereas KNS (temperate coniferous forests) had a higher value of specialization asymmetry. Our method offers an effective way for quickly comparing large and complex datasets from different ecosystem types. We identified key species among the four sites through plant–microbe networks, which provided an opportunity to understand how the behavior of core species affects other species. In addition, the results of the IDEN of plant–bacteria demonstrated the geographic distribution pattern of microorganisms. In short, the IDENP provides a novel perspective for exploring the relationships of interdomain species, and this study provides valuable insights into understanding coexistence between aboveground plants and belowground microorganisms, as well as guiding the management of forest ecosystems.

## Data Availability Statement

The datasets presented in this study can be found in online repositories. The name of the repository and accession number can be found below: National Center for Biotechnology Information (NCBI) BioProject, https://www.ncbi.nlm.nih.gov/bioproject/, PRJNA748146.

## Author Contributions

YZ conceived the study. YZ, YD, and KF contributed to the study design. WC and JY collected the soil samples and plant survey. WC conducted the data analysis and drafted the manuscript, figures, and tables. All authors contributed substantially to revisions.

## Conflict of Interest

The authors declare that the research was conducted in the absence of any commercial or financial relationships that could be construed as a potential conflict of interest. The reviewer WK declared a shared affiliation with several of the authors, YD and KF, to the handling editor at the time of review.

## Publisher’s Note

All claims expressed in this article are solely those of the authors and do not necessarily represent those of their affiliated organizations, or those of the publisher, the editors and the reviewers. Any product that may be evaluated in this article, or claim that may be made by its manufacturer, is not guaranteed or endorsed by the publisher.

## Footnotes

1
http://mem.rcees.ac.cn:8080

2
http://mem.rcees.ac.cn:8081
