# Supplementary material for: The Coexistence Relationship Between Plants and Soil Bacteria Based on Interdomain Ecological Network Analysis
Source: Front Microbiol. 2021 Dec 7;12:745582. doi: 10.3389/fmicb.2021.745582 (PMC8689066; doi:10.3389/fmicb.2021.745582)
Supplement: Supplementary file 1 [file Data_Sheet_1.docx]

Table S1 Comparison of observed network topological characteristics and random network topological structure. (KNS=Kanas, GS=Gansu, MLZ=Mulinzi, HN=Hainan)

|  | Observed network | Rewiring network | t | *P* |
| --- | --- | --- | --- | --- |
| KNS | | | | |
| Nestedness | 41.853 | 41.846±0.127 | -0.556 | 0.579 |
| Weighted nestedness | 0.481 | 0.465±0.006 | -24.633 | <0.001 |
| Specialisation asymmetry | 0.756 | 0.715±0.019 | -22.082 | <0.001 |
| C.score.HL | 0.879 | 0.882±0.017 | 1.787 | 0.077 |
| C.score.LL | 0.577 | 0.577±0.000 | 7.112 | <0.001 |
| Modularity (simulated annealing) | 0.570 | 0.568±0.002 | -13.602 | <0.001 |
| GS | | | | |
| Nestedness | 28.360 | 24.972±0.446 | -75.892 | <0.001 |
| Weighted nestedness | 0.380 | 0.451±0.008 | 94.873 | <0.001 |
| Specialisation asymmetry | 0.488 | 0.493±0.001 | 94.213 | <0.001 |
| C.score.HL | 0.735 | 0.701±0.003 | -104.825 | <0.001 |
| C.score.LL | 0.644 | 0.613±0.002 | -137.912 | <0.001 |
| Modularity (simulated annealing) | 0.353 | 0.318±0.006 | -62.457 | <0.001 |
| MLZ | | | | |
| Nestedness | 32.762 | 31.945±0.672 | -12.159 | <0.001 |
| Weighted nestedness | 0.381 | 0.406±0.011 | 23.783 | <0.001 |
| Specialisation asymmetry | 0.654 | 0.655±0.001 | 11.670 | <0.001 |
| C.score.HL | 0.616 | 0.577±0.007 | -59.370 | <0.001 |
| C.score.LL | 0.598 | 0.583±0.002 | -69.371 | <0.001 |
| Modularity (simulated annealing) | 0.382 | 0.362±0.010 | -20.906 | <0.001 |
| HN | | | | |
| Nestedness | 32.771 | 30.446±0.590 | -39.424 | <0.001 |
| Weighted nestedness | 0.190 | 0.249±0.011 | 55.327 | <0.001 |
| Specialisation asymmetry | 0.552 | 0.554±0.000 | 72.300 | <0.001 |
| C.score.HL | 0.769 | 0.748±0.002 | -110.165 | <0.001 |
| C.score.LL | 0.774 | 0.761±0.001 | -96.701 | <0.001 |
| Modularity (simulated annealing) | 0.413 | 0.357±0.009 | -61.247 | <0.001 |

Table S2 Plant, soil bacteria and node information of each module in four forest types. (KNS=Kanas, GS=Gansu, MLZ=Mulinzi, HN=Hainan)

|  | Plants | No. module | Number of OTU | Node degree | Zi | Pi | Network roles |
| --- | --- | --- | --- | --- | --- | --- | --- |
| KNS | *Betula pendula* | 1 | 9 | 9 | 3.00 | 0.00 | Module hubs |
|  | *Pinus sibirica* | 2 | 118 | 132 | 10.86 | 0.19 | Module hubs |
|  | *Larix sibirica* | 3 | 53 | 54 | 7.28 | 0.04 | Module hubs |
|  | *Picea obovata* | 4 | 133 | 147 | 11.53 | 0.18 | Module hubs |
| GS | *Corylus heterophylla* | 1 | 303 | 77 | 3.90 | 0.43 | Module hubs |
|  | *Lindera aggregata* |  |  | 127 | 7.83 | 0.23 | Module hubs |
|  | *Quercus aliena* |  |  | 273 | 12.55 | 0.52 | Module hubs |
|  | *Sorbus alnifolia* |  |  | 117 | 6.74 | 0.31 | Module hubs |
|  | *Viburnum betulifolium* |  |  | 107 | 4.78 | 0.54 | Module hubs |
|  | *Cotoneaster acuminatus* | 2 | 247 | 104 | 4.15 | 0.52 | Module hubs |
|  | *Crataegus kansuensis* |  |  | 260 | 9.62 | 0.57 | Module hubs |
|  | *Euonymus sanguineus* |  |  | 59 | 2.76 | 0.34 | Module hubs |
|  | *Pinus armandi* |  |  | 299 | 11.40 | 0.55 | Module hubs |
|  | *Acer mono* | 3 | 269 | 124 | 6.43 | 0.36 | Module hubs |
|  | *Acer tetramerum* |  |  | 446 | 12.89 | 0.67 | Network hubs |
|  | *Lindera obtusiloba* |  |  | 149 | 6.57 | 0.51 | Module hubs |
|  | *Swida hemsleyi* |  |  | 100 | 4.26 | 0.50 | Module hubs |
|  | *Cerasus polytricha* | 4 | 72 | 36 | 4.63 | 0.29 | Module hubs |
|  | *Lonicera ferdinandii* |  |  | 47 | 7.11 | 0.08 | Module hubs |
|  | *Malus hupehensis* | 5 | 24 | 28 | 4.90 | 0.26 | Module hubs |
| MLZ | *Symplocos anomala* | 1 | 254 | 240 | 13.02 | 0.27 | Module hubs |
|  | *Cyclobalanopsis multinervis* |  |  | 224 | 9.18 | 0.53 | Module hubs |
|  | *Quercus engleriana* | 2 | 37 | 40 | 6.08 | 0.14 | Module hubs |
|  | *Carpinus fargesiana* | 3 | 222 | 166 | 8.95 | 0.40 | Module hubs |
|  | *Eurya alata* |  |  | 148 | 6.68 | 0.51 | Module hubs |
|  | *Cornus kousa* |  |  | 134 | 8.21 | 0.24 | Module hubs |
|  | *Litsea elongata* |  |  | 88 | 4.70 | 0.36 | Module hubs |
|  | *Lindera obtusiloba* |  |  | 42 | 2.64 | 0.09 | Module hubs |
|  | *Cyclobalanopsis myrsinaefolia* | 4 | 58 | 62 | 7.62 | 0.12 | Module hubs |
| HN | *Beilschmiedia laevis* | 1 | 163 | 26 | 1.63 | 0.42 | Peripheral species |
|  | *Cryptocarya chingii* |  |  | 88 | 7.92 | 0.17 | Module hubs |
|  | *Ervatamia officinalis* |  |  | 68 | 5.03 | 0.39 | Module hubs |
|  | *Neolitsea ellipsoidea* |  |  | 131 | 7.92 | 0.56 | Module hubs |
|  | *Walsura robusta* |  |  | 39 | 3.08 | 0.27 | Module hubs |
|  | *Ardisia quinquegona* | 2 | 74 | 45 | 4.37 | 0.44 | Module hubs |
|  | *Gironniera subaequalis* |  |  | 65 | 7.41 | 0.36 | Module hubs |
|  | *Epiprinus siletianus* | 3 | 226 | 65 | 3.25 | 0.38 | Module hubs |
|  | *Litsea baviensis* |  |  | 125 | 6.03 | 0.46 | Module hubs |
|  | *Mallotus hookerianus* |  |  | 121 | 5.82 | 0.46 | Module hubs |
|  | *Microcos chungii* |  |  | 145 | 7.68 | 0.38 | Module hubs |
|  | *Polyalthia lauii* |  |  | 178 | 8.39 | 0.49 | Module hubs |
|  | *Saprosma merrillii* |  |  | 76 | 3.82 | 0.39 | Module hubs |
|  | *Aidia oxyodonta* | 4 | 184 | 46 | 3.55 | 0.42 | Module hubs |
|  | *Blastus cochinchinensis* |  |  | 71 | 6.32 | 0.32 | Module hubs |
|  | *Diospyros cathayensis* |  |  | 44 | 3.31 | 0.43 | Module hubs |
|  | *Memecylon ligustrifolium* |  |  | 99 | 6.90 | 0.53 | Module hubs |
|  | *Prismatomeris connata* |  |  | 79 | 4.82 | 0.58 | Module hubs |
|  | *Psychotria rubra* |  |  | 66 | 5.75 | 0.34 | Module hubs |
|  | *Xanthophyllum hainanense* |  |  | 68 | 3.89 | 0.57 | Module hubs |


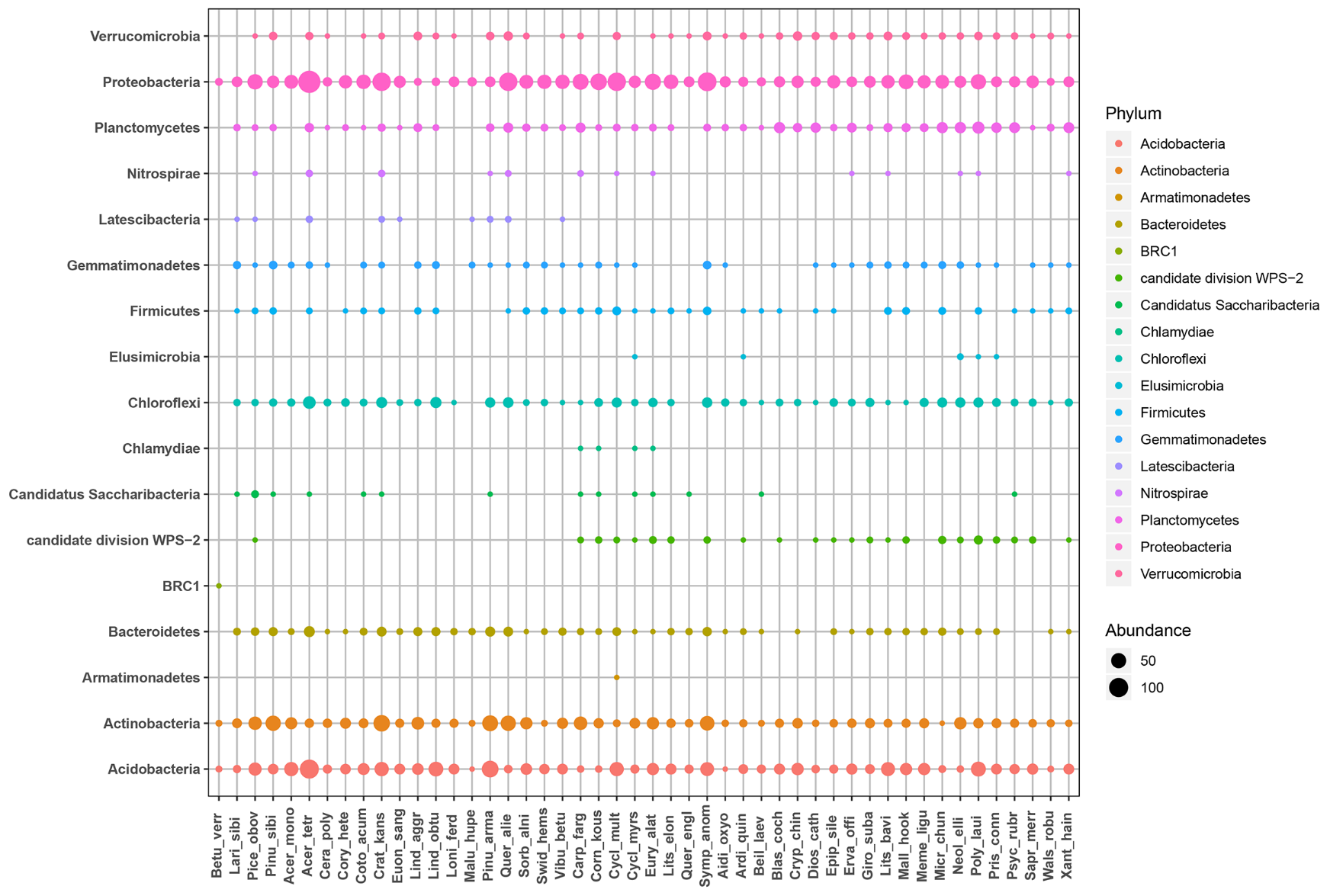
Figure S1 Abundance of soil bacterial phyla associated with plants (KNS=Kanas, GS=Gansu, MLZ=Mulinzi, HN=Hainan).


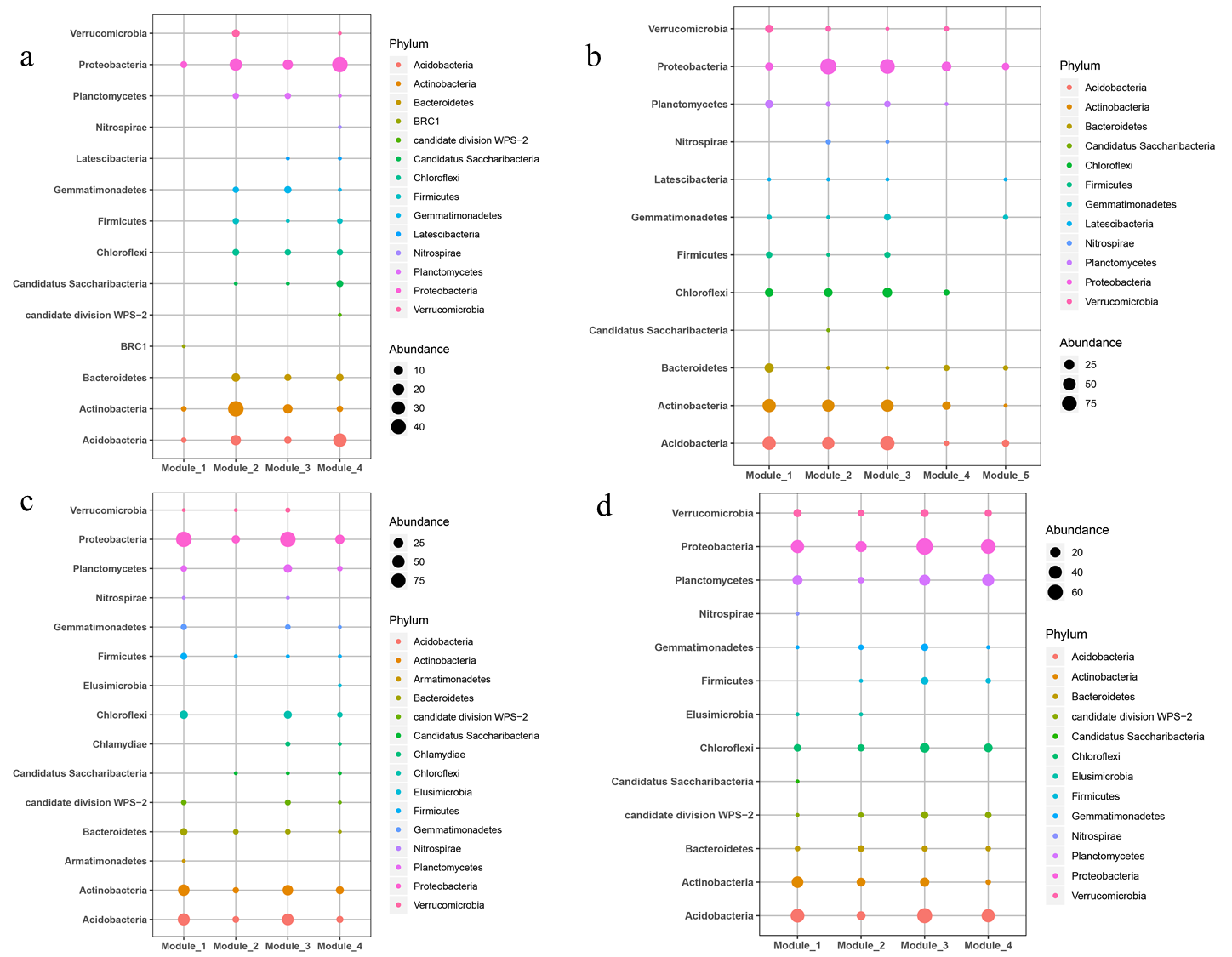
Figure S2 Community composition of soil bacteria within each module for the observed IDEN at phylum level. a) Kanas (KNS); b) Gansu (GS); c) Mulinzi (MLZ); d) Hainan (HN).
